# Two Cases of Sloughing Ulceration, in Which Nitric Acid Was the Principal Local Application

**Published:** 1826-08

**Authors:** 

**Affiliations:** St. Thomas's Hospital


*Two Cases of Sloughing Ulceration, in which Nitric Acid was the principal local application.* Treated at St. Thomas's Hospital, by Mr. Green.

I. *Case of Sloughing Ulceration of the Labium and Nates, which terminated favourably.*

June 3d, 1824.—Mary Ann Welch, ætatis eighteen, of delicate constitution, though her health is said to have been good; has been in the habit of drinking spirits. About six weeks ago, she had a severe attack of gonorrhœa: the discharge at first was of a greenish yellow colour, and thick, but exceedingly irritating, with micturition and scalding. An ulcer followed on the left labium. She had no medical aid, but kept her bowels regular, which gave her great relief. The ulceration of the labium commenced about a fortnight after the appearance of the discharge: it has never given her any pain. She has a sore upon the left nates, which first appeared spontaneously about two years since, whilst she was labouring under a discharge: it was then cured by the application of black wash, and rubbing-in Unguentum Hydrargyri to ptyalism. She says that this sore reappeared seven weeks ago. It now presents a cupped, dark, and mottled appearance, with a hard deep-red base, and is of the circumference of a teacup. The discharge from it is dark and bloody, having an offensive smell.

June 15th.—Simple remedies having been used without success, the strong nitric acid was applied to the sore yesterday, by means of a circular piece of lint dipped in it. It occasioned considerable pain, but she bore it well; and an opiate was given, which produced sleep, after which great relief was experienced.

18th.—Scarcely any discharge from labium, which is dressed with dry lint. Cat. Lini applied to sore on nates, and nitric acid wash, occasionally. Health greatly improved.

21st.—Pulse eighty-six, small; tongue white; bowels open. Sore has lost its dark appearance, and now looks very healthy. Nitric acid lotion gives burning pain for five minutes after it is applied.

July 22d.—Has continued to improve progressively. All the sores now healed.

II. *Fatal Case of Sloughing of the Labia, &c.*

November 19th, 1824.—Mary Williams, aged fifteen, of florid complexion; has previously enjoyed good health, but her constitution appears delicate; says she has not been *particularly* addicted to the use of spirits. The upper and inner part of the right labium has sloughed to a considerable extent, as well as a corresponding portion of the left labium; together with the nymphæ and præputium clitoridis. A considerable portion of the under part of both labia is likewise lost; and the ulceration has even extended to the perineum and nates. The ulcerated parts have a dark appearance, discharging dark bloody matter; and the surrounding integuments are red and painful. There is a good deal of burning pain experienced in the parts, with troublesome itching and shooting, especially about the anus. She suffers considerably whenever she has a motion; and, on making water, bleeding takes place. She is extremely weak, and entirely confined to bed. Sleeps badly. Pulse 120, small and jerking; appetite very bad; tongue coated with yellow fur; bowels open by medicine. When asleep, perspires profusely, but otherwise her skin is burning hot and dry. Urine high coloured. Menstruated a week ago, but up to that time had not been regular for five months.

About two months ago she observed a discharge from the pudenda, but without scalding. About two weeks afterwards, she felt considerable itching of the labia and perineum. A hard red lump formed in the genital fissure, which itched, and, after being scratched, ulcerated. On her treating the labia in the same way, small pimples were produced, which soon became sores, and quickly deepened. About three weeks ago, these sores began to spread, and to excite burning pain; they had a dark appearance, and were covered with clots. At this time she was attacked with rigors, which lasted a few minutes, and terminated in sweating. She has taken pills, which did not affect her mouth, and applied lotions to the parts, previous to her admission, which however afforded no relief.

P. Rhæi c. Hyd. Submur. gr. xij. statim.—P. Ipecac. C. gr. viij. o. n.—Mist. Potas. Cit. c. Tr. Opii, m. v. quartis horis.—Hirudines x. pubi.—Acid. Nit. Fort. ulceribus.

Nov. 21st.—Nitric acid applied by means of a camel's-hair brush. The pain was not very severe at the time, but, about ten minutes afterwards, considerable smarting and burning pain was felt.

26th.—Sloughs extending. An ulcer, of the size of half-a-crown, on left groin, which has a dark mottled appearance.

℞. Pil. Opii, gr. i. sextis horis.—Acid. Nit. Fort.

December 1st.—Deep ulceration, with sloughing, of the size of a crown-piece, discovered on the lower part of sacrum. Considerable excoriation extending from it along the fossa of nates.

Vini Rubri, ℥ iv. quotidie.—Turpentine dressing to be applied after the wash. Cat. Dauci ulceri super sacrum.—Extra meat.

3d.—Bowels purged. Pain of sore relieved.

Omitt. Mist. Potassæ et P. Ipec. C.—Repet. alia.

5th.—Sores look better, and there is less surrounding redness.

Porter ℔ i., Brandy ʒvj. daily.—Arrow-root.—Wine and Sago to be omitted.

13th.—Several deep ulcers from pressure, one on each trochanter, and one on right ileum.

Lotio Plumbi.—Cat. Lini.—Repet. medicamenta, &c.

January 8th, 1825.—All the sores looking better. Motions passed only at night, and then involuntarily. Pulse 128, small and fluttering.

14th.—Has eaten nothing since the 9th. Refuses brandy; takes beef-tea and porter. Sore on sacrum has filled up, having been strapped with equal parts of Emp. Plumb. et Emp. Sapon.; but there is a dark spot in the centre.

Acid. Nit. dil. m. x. ex Aquæ ℥ iij.—Syrupi q. s. ter die.—Porter ℔ i.

18th.—A fistulous opening, discharging dark thin pus, in right groin. An ulcer, the size of palm of the hand, on right ileum. Ecchymosis on ribs and extremities. Takes her porter and four eggs daily.

Emplast. Plumbi.—Repet. alia.

26th.—Gradually sunk, and died this day in great pain.

